# Supplementary material for: Striatal Acetylcholine Dip-Rebound Is Induced by Direct-Pathway Neurons and Encode Action-Outcome Contingency
Source: bioRxiv. 2026 Jan 14:2026.01.13.699373. Preprint. [Version 1] doi: 10.64898/2026.01.13.699373 (PMC12871142; doi:10.64898/2026.01.13.699373)
Supplement: Supplement 1 [file NIHPP2026.01.13.699373v1-supplement-1.pdf]

bioRxiv preprint doi: https://doi.org/10.64898/2026.01.13.699373; this version posted January 14, 2026. The copyright holder for this preprint (which was not certified by peer review) is the author/funder, who has granted bioRxiv a license to display the preprint in perpetuity. It is made available under aCC-BY 4.0 International license.

Supplementary

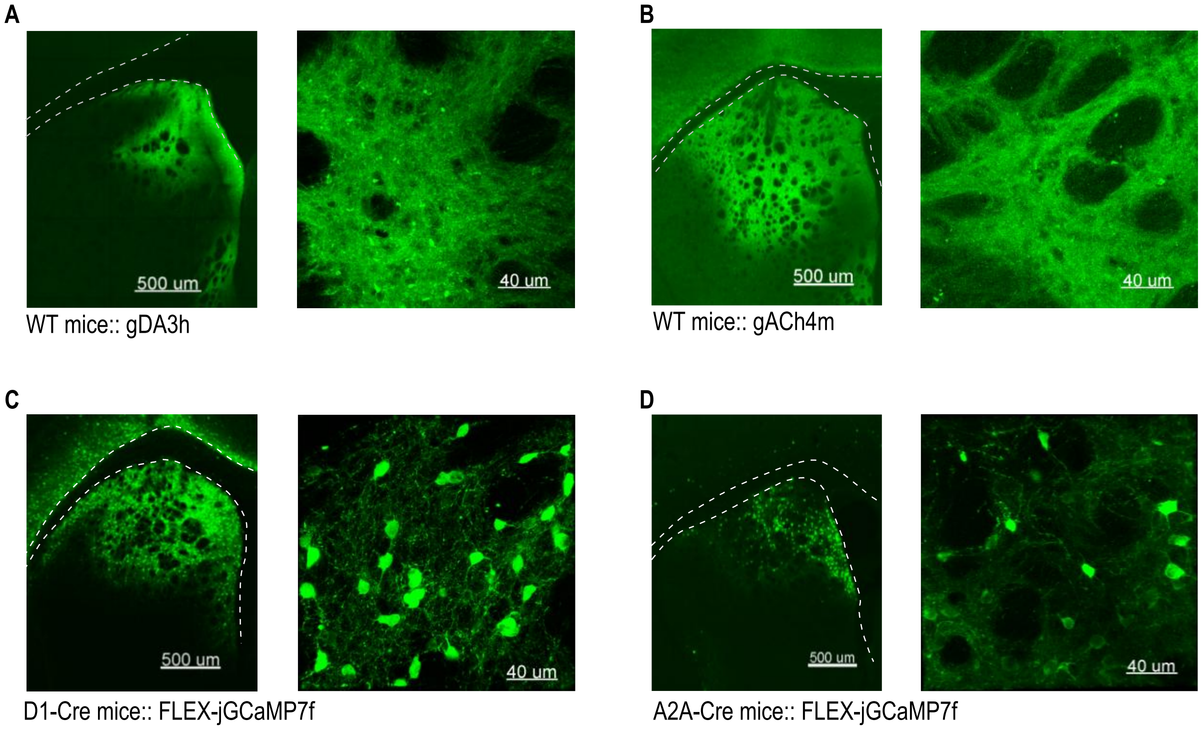

Supplementary Figure 1. Histology for gACh4m, gDA3h, and jGCaMP7f expression in D1-Cre mice or A2A-Cre mice in the DMS.

A. Confocal images of gACh4m expression for a representative mouse recorded for Fig.1. The Left image shows the expression of gACh in the DMS. The right image shows the enlarged detail of gACh expression.

B. Confocal images of gDA3h expression for a representative mouse recorded for Fig.1. The Left image shows the expression of gDA3h in the DMS. The right image shows the enlarged detail of gDA3h expression.

C. Confocal images of Cre-dependent jGCaMP7f expression for a representative D1-Cre mouse recorded for Fig.1. The Left image shows the expression of jGCaMP7f in the DMS. The right image shows the enlarged detail of jGCaMP7f expression.

D. Confocal images of Cre-dependent jGCaMP7f expression for a representative A2A-Cre mouse recorded for Fig.1. The Left image shows the expression of jGCaMP7f in the DMS. The right image shows the enlarged detail of jGCaMP7f expression.

bioRxiv preprint doi: https://doi.org/10.64898/2026.01.13.699373; this version posted January 14, 2026. The copyright holder for this preprint (which was not certified by peer review) is the author/funder, who has granted bioRxiv a license to display the preprint in perpetuity. It is made available under aCC-BY 4.0 International license.

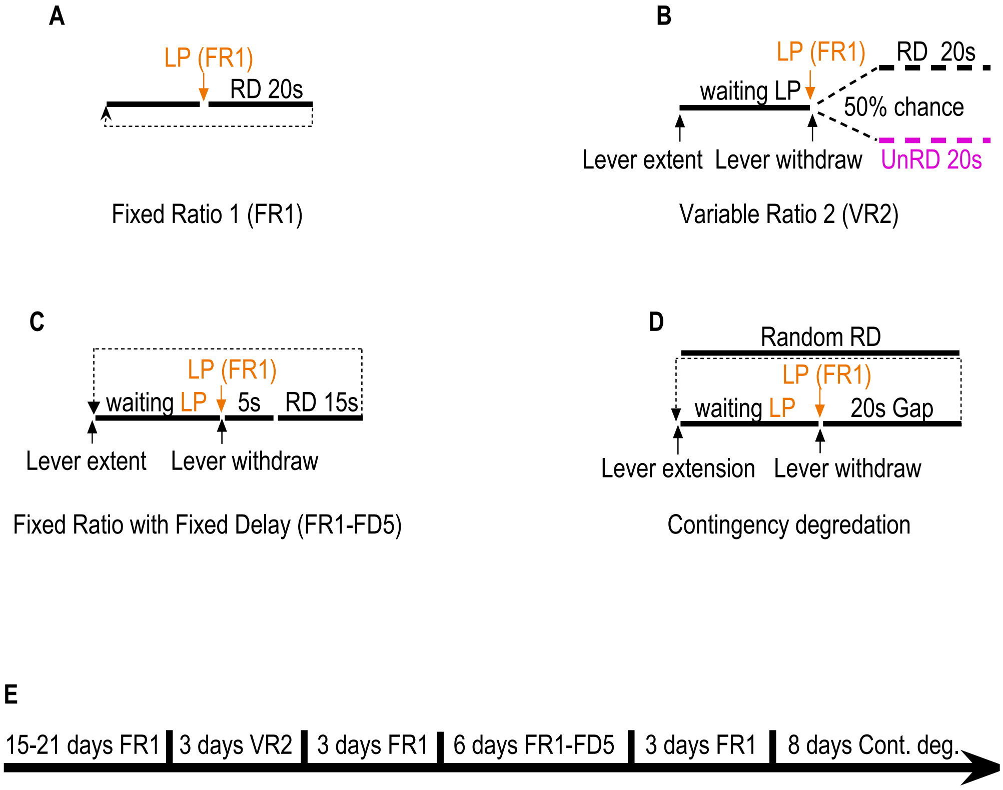

Supplementary Figure 2. Behavioral task schedules and training timeline

(**A–D**) Schematic diagrams of the four operant conditioning schedules used across training phases. More details can be found in the Method section

A. Fixed Ratio 1 (FR1): A single lever press (LP) after lever extension (L extent) triggered immediate reward delivery (RD; 20 s timeout).

B. Variable Ratio 2 (VR2): Each lever press had a 50% probability of resulting in reward delivery (RD; 10 s timeout) or no reward (non-RD; 10 s timeout).

C. Fixed Ratio with Fixed Delay (FR1–FD5): A single lever press initiated a fixed 5-second delay, after which the reward was delivered (RD; 10 s timeout).

D. Fixed Ratio with Variable Interval (FR1–VI80): lever presses initiated a 10-second timeout. Reward was delivered with a variable time interval (~80 s).

E. Timeline of the behavioral training protocol. Animals were initially trained under the FR1 schedule for 15–21 days, followed sequentially by VR2 (5 days), FR1–FD5 (6 days), a brief reversion to FR1 (4 days), and finally FR1–VI80 (8 days).

bioRxiv preprint doi: https://doi.org/10.64898/2026.01.13.699373; this version posted January 14, 2026. The copyright holder for this preprint (which was not certified by peer review) is the author/funder, who has granted bioRxiv a license to display the preprint in perpetuity. It is made available under aCC-BY 4.0 International license.

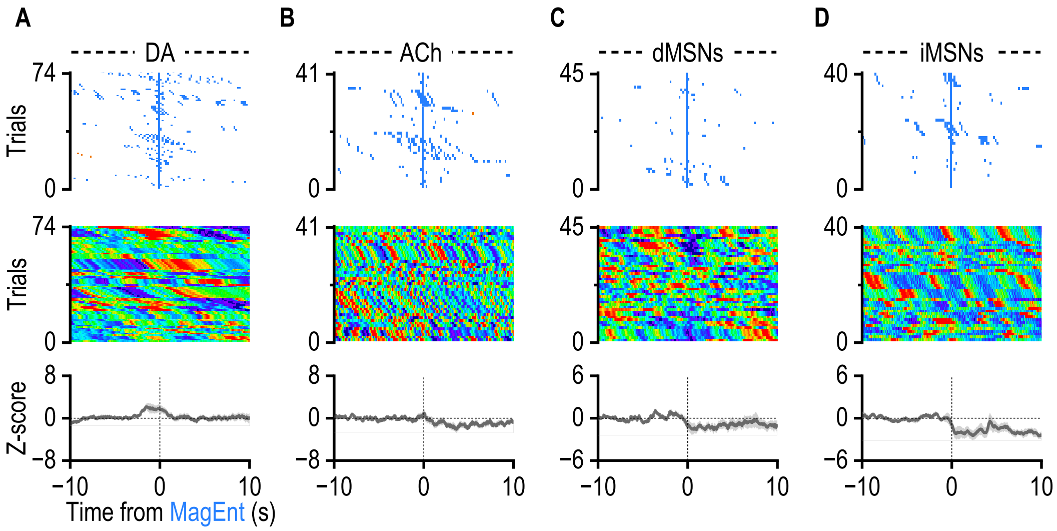

Supplementary Figure 3. ACh, DA, dMSN, and iMSN have no significant response to magazine entry in naïve mice.

A-D. (Top) Sample Raster plots showing the distribution of lever presses and magazine entries in naïve mice from 4 groups(1, wild-type mice infused with AAV-gACh; 2, wild-type mice infused with AAV-gDA; 3, D1-Cre mice infused with AAV-Flex-jGCaMP7f; 4, A2A-Cre mice infused with AAV-Flex-jGCaMP7f) aligned to magazine entries. (Middle) Sample heatmap showing neuronal activity or neurotransmitter dynamics in the same session as Top, aligned to the magazine entries. (Bottom) average neuronal activity or neurotransmitter dynamics of four groups aligned to magazine entries.

bioRxiv preprint doi: https://doi.org/10.64898/2026.01.13.699373; this version posted January 14, 2026. The copyright holder for this preprint (which was not certified by peer review) is the author/funder, who has granted bioRxiv a license to display the preprint in perpetuity. It is made available under aCC-BY 4.0 International license.

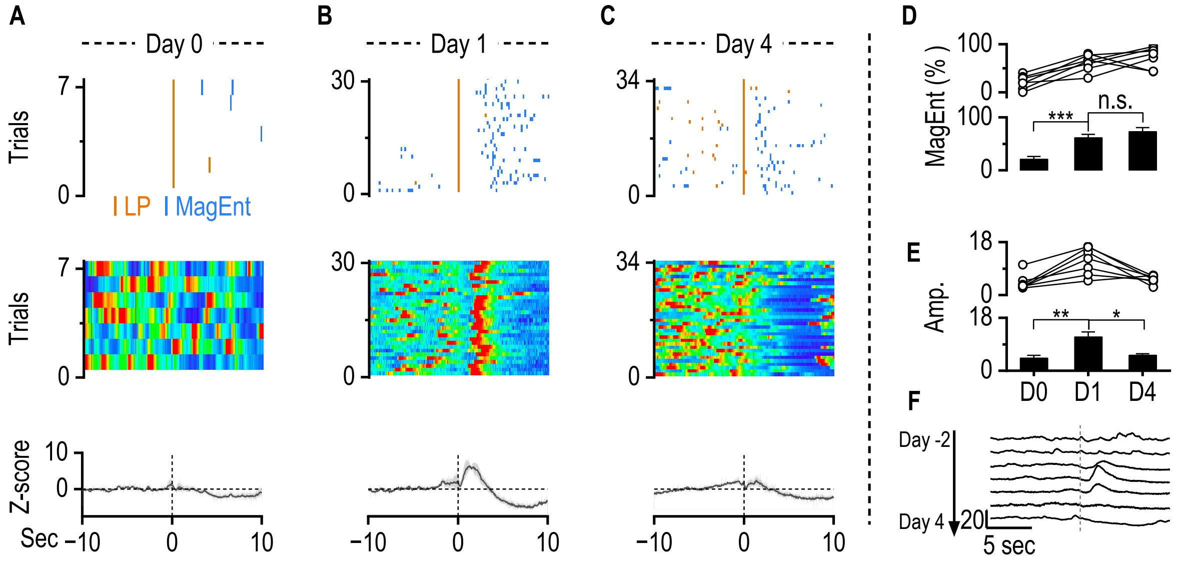

Supplementary Figure 4. iMSN activity emerges during initial contingency learning and diminishes with continued training

At the beginning of FR1 training, before the animals had learned the action-outcome contingency, lever presses did not evoke any detectable iMSN activity. However, after several days of training, during the initial phase when mice first learned the contingency, iMSNs showed increased activity both before and after lever presses. With continued training over the following four days, this iMSN activity gradually diminished. (A–C) Representative behavioral and photometry data from a sample A2A-Cre mouse expressing jGCaMP7f in the DMS and trained under an FR1 schedule. Top: raster plots showing lever presses (orange) and magazine entries (blue) across trials. Middle: trial-aligned heatmaps of iMSN calcium signals time-locked to lever press (time = 0). Bottom: population average iMSN activity (Z-scored ΔF/F) across 7 mice.

A. Day 0: prior to learning, lever presses were not consistently followed by immediate magazine entries, and iMSNs showed no activity change.

B. Day 1: the first day the mouse exhibited learned contingency between lever press and reward; magazine entries reliably followed lever presses, and iMSNs showed increased activity around lever presses.

bioRxiv preprint doi: https://doi.org/10.64898/2026.01.13.699373; this version posted January 14, 2026. The copyright holder for this preprint (which was not certified by peer review) is the author/funder, who has granted bioRxiv a license to display the preprint in perpetuity. It is made available under aCC-BY 4.0 International license.

C. Day 4: While immediate magazine entries persisted, iMSN activity following lever presses was reduced.

D. Quantification of the percentage of trials with immediate magazine entries (within 2 s after lever press) shows significant increases on Day 1 and Day 4 compared to Day 0 (n = 7 mice; Day 0 vs Day 1: $t_{(6)} = 6.09$, ***$p < 0.001$; Day 1 vs Day 4: $t_{(6)} = 1.00$, $p > 0.05$, paired t test).

E. Amplitude of iMSN activity following lever press was significantly elevated on Day 1 compared to Day 0 and Day 4 (Day 0 vs Day 1: $t_{(6)} = 4.71$, **$p < 0.01$; Day 1 vs Day 4: $t_{(6)} = 3.22$, *$p < 0.05$, paired t test).

F. Example continuous trace of iMSN activity from one mouse across multiple training days (Day -2 to Day 4), showing transient activation emerging on the day of learning and declining thereafter.

bioRxiv preprint doi: https://doi.org/10.64898/2026.01.13.699373; this version posted January 14, 2026. The copyright holder for this preprint (which was not certified by peer review) is the author/funder, who has granted bioRxiv a license to display the preprint in perpetuity. It is made available under aCC-BY 4.0 International license.

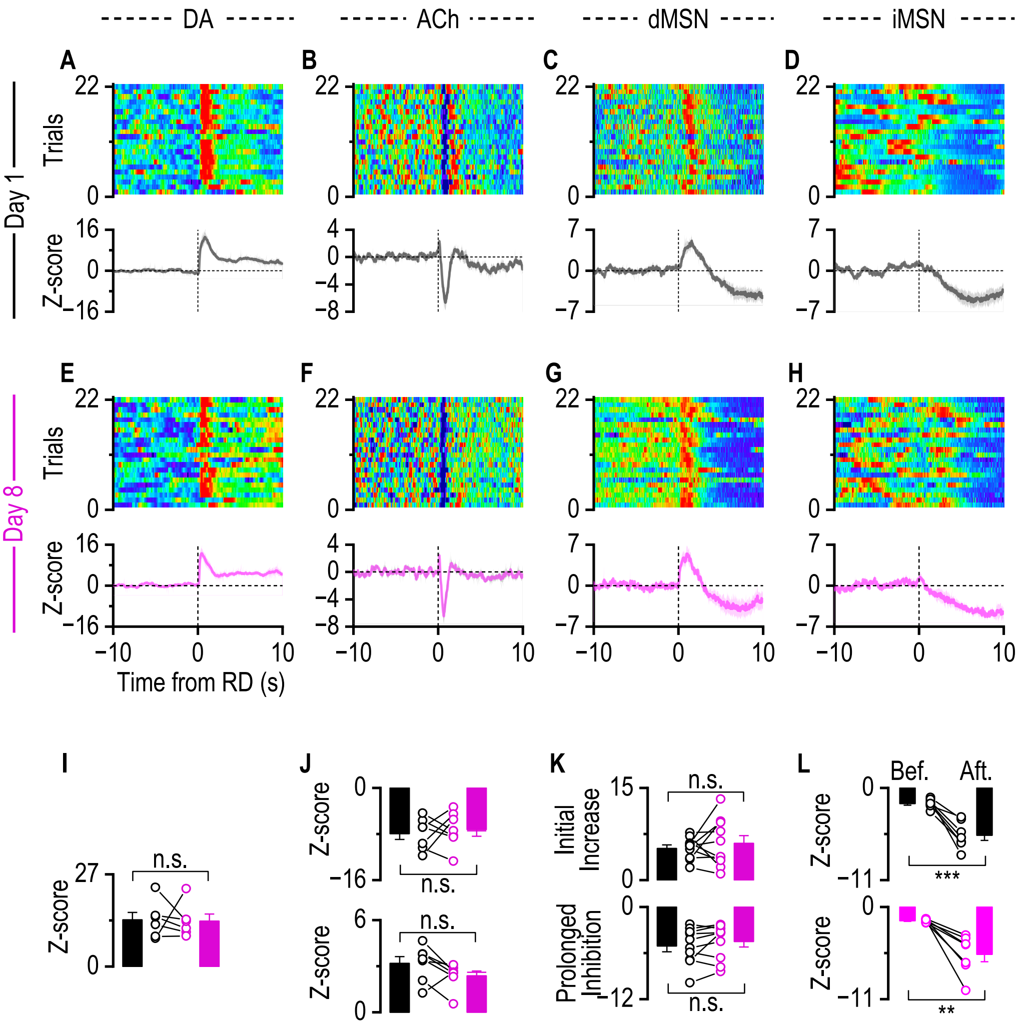

Supplementary Figure 5. Contingency degradation does not change striatal ACh, DA, dMSN, and iMSN response to reward delivery.

Mice underwent an 8-day contingency degradation protocol in which lever presses no longer led to reward delivery; instead, rewards were delivered non-contingently at ~80-second variable intervals. (A–D) Neural dynamics on Day 1 of contingency degradation training.

A. Top: Heatmap of ACh signals across trials aligned to reward delivery (time 0) showing a brief burst followed by a dip-rebound pattern. Bottom: Average ACh response.

B. Top: Heatmap of DA signals showing a robust increase following reward delivery. Bottom: Average DA response.

bioRxiv preprint doi: https://doi.org/10.64898/2026.01.13.699373; this version posted January 14, 2026. The copyright holder for this preprint (which was not certified by peer review) is the author/funder, who has granted bioRxiv a license to display the preprint in perpetuity. It is made available under aCC-BY 4.0 International license.

C. Top: Heatmap of dMSN calcium activity showing an increase followed by a sustained decrease after reward delivery. Bottom: Average dMSN response.

D. Top: Heatmap of iMSN calcium activity showing a reduction following reward delivery. Bottom: Average iMSN response.

E–H. Same as panel **A–D**, but for Day 8 of contingency degradation training. DA, ACh, dMSN, and iMSN responses remained qualitatively similar to Day 1, indicating persistent neural responses to reward delivery.

I–L. Quantification of signal amplitudes for DA increase (**I**), ACh dip and rebound (**J**), and MSN activity (**K**, **L**) on Day 1 (black) and Day 8 (magenta), showing no significant difference across days (DA: $t_{(5)} = 0.10$, $p > 0.05$, paired t test; ACh dip: $t_{(6)} = 0.47$, $p > 0.05$, paired t test; ACh rebound: $t_{(6)} = 1.98$, $p > 0.05$, paired t test; dMSN initial activity increase: $t_{(9)} = 0.80$, $p > 0.05$, paired t test; dMSN prolonged inhibition: $t_{(9)} = 1.41$, $p > 0.05$, paired t test; iMSN: Day 1 before RD vs. after RD, $t_{(6)} = 6.50$, ***$p < 0.001$, paired t test; Day 8 before RD vs. after RD, $t_{(6)} = 4.47$, **$p < 0.01$, paired t test;).

bioRxiv preprint doi: https://doi.org/10.64898/2026.01.13.699373; this version posted January 14, 2026. The copyright holder for this preprint (which was not certified by peer review) is the author/funder, who has granted bioRxiv a license to display the preprint in perpetuity. It is made available under aCC-BY 4.0 International license.

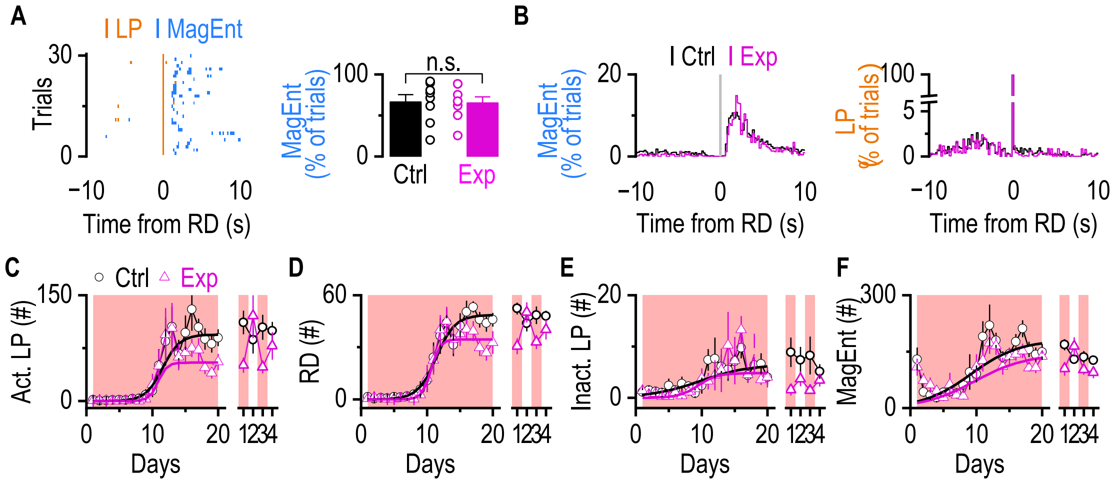

Supplementary Figure 6. dMSN inhibition after lever press decreased the reinforcing effect

To test the functional role of dMSN activity after reward delivery in instrumental learning, and to verify that the optical inhibition of dMSN is sufficient to diminish the ACh dip-rebound. D1-Cre mice infused with DIO-ChRmine and gACh sensor underwent an FR1 schedule with a 100% chance of laser delivery after reward delivery.

A. Representative raster plots showing active lever presses and magazine entries aligned to reward delivery (Left). Immediate magazine entries after reward deliveries (within 3 seconds) did not differ between the control and experimental groups (Right). n.s., not significant, $p > 0.05$ by unpaired t-test, n = 8 (Ctrl) and 8 (Exp) mice.

B. Average distribution of Magazine entry (top) and lever presses(bottom) between Ctrl (black) and Exp (red) group.

C. The Exp group showed higher maximal Active lever pressing (Act. LP) than the Ctrl group, despite similar acquisition dynamics. Solid curves represent the best-fit 3-parameter logistic regression (Ctrl group: $K = 94.10 \pm 5.76$, $x_0 = 11.57 \pm 0.59$, $k = 0.77 \pm 0.12$, ($R^2 = 0.89$); Exp group: $K = 54.40 \pm 4.39$, $x_0 = 10.87 \pm 0.81$, $k = 1.50 \pm 0.64$, ($R^2 = 0.73$)). Wald z-tests indicated significant group differences in asymptotic performance (K), while the inflection point (x_0) and slope (k) did not differ ($z_{x0} = -0.70$, $p > 0.05$; $z_k = 1.13$, $p > 0.05$; z_K =

bioRxiv preprint doi: https://doi.org/10.64898/2026.01.13.699373; this version posted January 14, 2026. The copyright holder for this preprint (which was not certified by peer review) is the author/funder, who has granted bioRxiv a license to display the preprint in perpetuity. It is made available under aCC-BY 4.0 International license.

-5.48, ***p < 0.001). On Day 22 and Day 24, the laser will be removed. There is no difference in Act. LP between Laser off sessions and Laser on sessions in the Ctrl group, but in the experimental group, removing the laser stimulation significantly increased Act. LP (Ctrl group: $t_{(7)}$ = 0.25, p >0.05, paired t test; Exp group: $t_{(6)}$ = 2.6, *p <0.05, paired t test).

D. The Exp group showed higher maximal reward delivery (RD) than the Ctrl group despite similar acquisition dynamics (Ctrl group: K = 48.59 ± 1.89, x_0 = 11.43 ± 0.46, k = 0.71 ± 0.09, (R^2 = 0.96); Exp group: K = 34.48 ± 1.81, x_0 = 10.62 ± 0.39, k = 1.50 ± 0.37, (R^2 = 0.94)). Wald z-tests indicated significant group differences in asymptotic performance (K), while the inflection point (x_0) and slope (k) did not differ (z_{x0} = -1.35, p > 0.05; z_k = 2.10, p > 0.05; z_K = -5.39, ***p < 0.001). Same as Act. LP, there is no difference in RD between Laser off sessions and Laser on sessions in the Ctrl group, but in the experimental group, removing the laser stimulation significantly increased Act. LP (Ctrl group: $t_{(7)}$ = 0.09, p >0.05, paired t test; Exp group: $t_{(6)}$ = 2.78, *p <0.05, paired t test).

E. Inactive lever pressing (Inact. LP) in the Ctrl group and Exp group over sessions did not follow a clear sigmoidal trend (Ctrl group: K = 6.57 ± 1.64, x_0 = 9.82 ± 2.95, k = 0.26 ± 0.13, (R^2 = 0.56); Exp group: K = 6.63 ± 4.53, x_0 = 13.28 ± 8.37, k = 0.23 ± 0.17, (R^2 = 0.33)).

F. The number of Magazine entries (MagEnt) in the Ctrl group and Exp group over sessions did not follow a clear sigmoidal trend (Ctrl group: K = 180.94 ± 28.59, x_0 = 9.21 ± 1.95, k = 0.27 ± 0.08, (R^2 = 0.54); Exp group: K = 143.20 ± 28.79, x_0 = 9.79 ± 2.72, k = 0.26 ± 0.14, (R^2 = 0.18)).

bioRxiv preprint doi: https://doi.org/10.64898/2026.01.13.699373; this version posted January 14, 2026. The copyright holder for this preprint (which was not certified by peer review) is the author/funder, who has granted bioRxiv a license to display the preprint in perpetuity. It is made available under aCC-BY 4.0 International license.

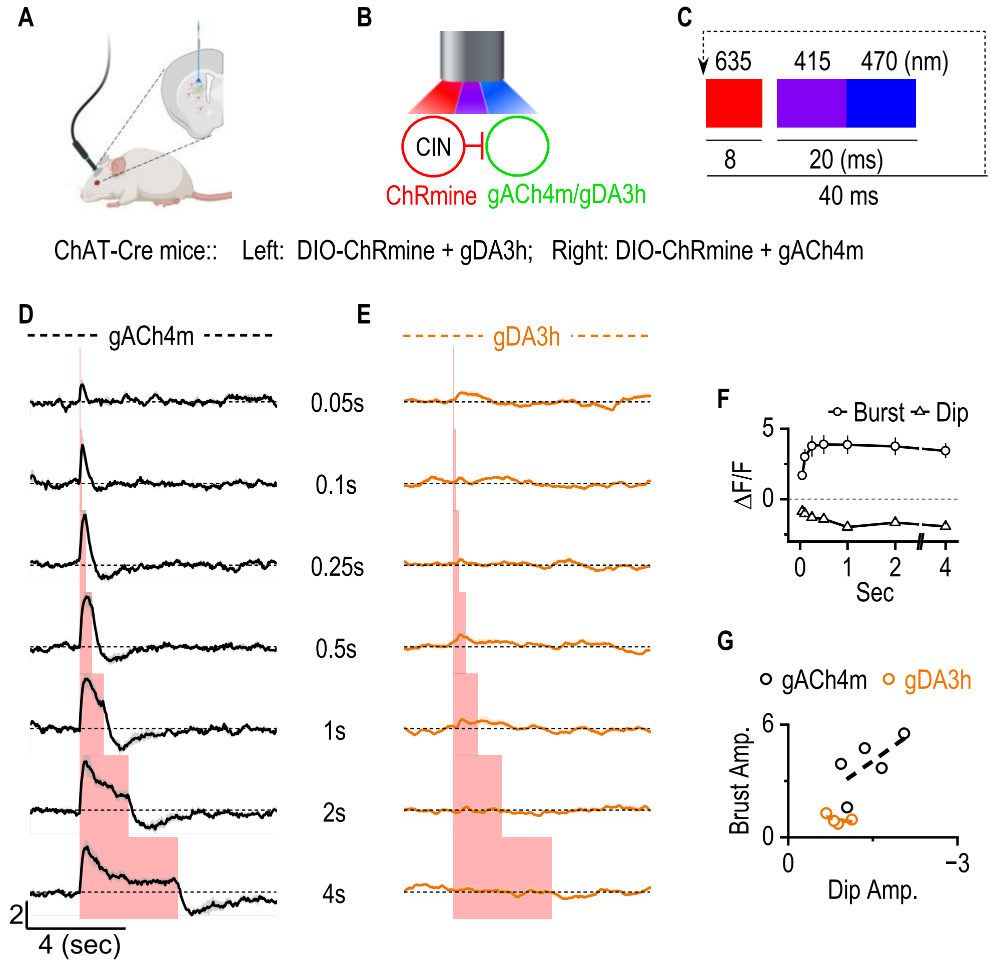

Supplementary Figure 7. Optical stimulation of CIN induced an ACh burst-dip in vivo and no response in DA release.

A. Diagram of the combined experimental setup for ChAT-Cre mice, showing simultaneous optogenetic stimulation and fiber photometry to assess DA release, as a control, or ACh release, as the experimental group, following CIN activation. In the control group, ChAT-Cre mice were infused with AAV-DIO ChRmine and AAV-gACh4m. In the Experimental group, ChAT-Cre mice were infused with AAV-DIO-ChRmine and AAV-gDA3h.

B. Simplified circuit diagram depicting Cholinergic input from CIN onto other neurons expressing $GRAB_{gACh4m}$ or $GRAB_{gDA3h}$.

C. Stimulation protocol: A cyclic sequence of three excitation wavelengths was used—635 nm (8 ms) for optogenetic activation of ChRmine triggered by TTL input, 415 nm (10 ms) as a

bioRxiv preprint doi: https://doi.org/10.64898/2026.01.13.699373; this version posted January 14, 2026. The copyright holder for this preprint (which was not certified by peer review) is the author/funder, who has granted bioRxiv a license to display the preprint in perpetuity. It is made available under aCC-BY 4.0 International license.

photometry reference, and 470 nm (10 ms) for excitation of gACh4m. The full cycle lasted 40 ms and was continuously repeated to enable simultaneous optogenetic stimulation and real-time monitoring of ACh dynamics via fiber photometry.

D. In vivo fiber photometry traces of ACh dynamics during optogenetic stimulation of CIN at increasing durations (0.05, 0.1, 0.25, 0.5, 1, 2, and 4s). ACh burst and dip become more pronounced with longer CIN stimulation (n = 5 mice).

E. In vivo fiber photometry traces of DA dynamics during optogenetic stimulation of CIN at increasing durations (0.05, 0.1, 0.25, 0.5, 1, 2, and 4s). DA has no response to CIN activation, implying that even though DA terminals express nicotinic receptors, ACh dynamics did not affect DA release in vivo (n = 4 mice).

F. Quantification of ACh Burst and dip amplitudes evoked by optogenetic stimulation of CIN at varying durations. The burst and dip amplitudes increased with stimulation duration and peaked at 0.5 seconds.

G. A negative correlation between ACh burst and dip amplitudes was observed following CIN stimulation (r = -0.66, R^2 = 0.44, slope = -2.13). Each dot represents one mouse in response to 25Hz, 0.5-second stimulation.

bioRxiv preprint doi: https://doi.org/10.64898/2026.01.13.699373; this version posted January 14, 2026. The copyright holder for this preprint (which was not certified by peer review) is the author/funder, who has granted bioRxiv a license to display the preprint in perpetuity. It is made available under aCC-BY 4.0 International license.

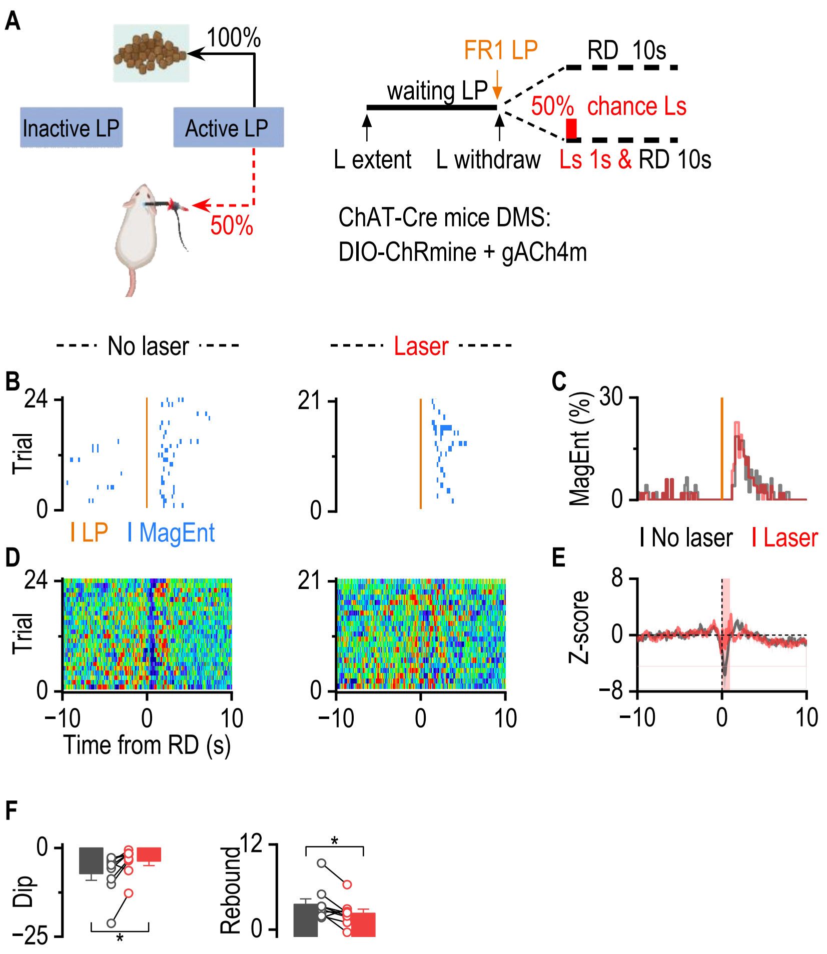

Supplementary Figure 8. Optical stimulation of CIN after reward delivery is sufficient to disrupt ACh dip-rebound.

To verify that the optical stimulation of CIN is sufficient to diminish the ACh dip-rebound. Well-trained ChAT-Cre mice infused with DIO-ChRmine and gACh4m und a FR1 schedule with a 50% chance of laser delivery after reward delivery.

A. Schematic of the behavioral paradigm. Mice were tested under an FR1 schedule in which each lever press (LP) was followed by reward delivery (RD; 100% probability) and, in 50% of trials, a 1-s 5 Hz red laser stimulation (Ls) to optogenetically activate CINs and disrupt the ACh dip–rebound.

B. Example raster plots of LPs (orange ticks) and magazine entries (MagEnt; blue ticks) in control (no-laser) trials (left) and laser trials (right).

bioRxiv preprint doi: https://doi.org/10.64898/2026.01.13.699373; this version posted January 14, 2026. The copyright holder for this preprint (which was not certified by peer review) is the author/funder, who has granted bioRxiv a license to display the preprint in perpetuity. It is made available under aCC-BY 4.0 International license.

C. Average distribution of MagEnt relative to LP onset for control (gray) and laser (red) trials (n = 6 mice), showing similar timing of reward collection between conditions.

D. Heatmaps of z-scored ACh dynamics aligned to RD for control (left) and laser (right) trials.

E. Trial-averaged ACh dynamics (mean ± SEM) showing that laser stimulation abolished the dip–rebound pattern (n = 9 mice).

F. Statistics show that the 1-second 5 Hz laser delivery after reward delivery diminished the amplitude of the ACh dip-rebound after reward delivery (Dip: $t_{(8)} = 3.26$, *$p < 0.05$, paired t test; Rebound: $t_{(8)} = 2.77$, *$p < 0.05$, paired t test).
